# Interval between secondary cytoreductive surgery and adjuvant chemotherapy is not associated with survivals in patients with recurrent ovarian cancer

**DOI:** 10.1186/s13048-019-0602-5

**Published:** 2019-12-31

**Authors:** Soo Young Jeong, Chel Hun Choi, Tae Joong Kim, Jeong Won Lee, Byoung-Gie Kim, Duk Soo Bae, Yoo-Young Lee

**Affiliations:** Division of Gynecologic Oncology, Departments of Obstetrics and Gynecology, Samsung Medical Center, Sungkyunkwan University School of Medicine, 81 Irwon-ro, Gangnam-gu, Seoul, 06351 Republic of Korea

**Keywords:** Secondary cytoreductive surgery, Adjuvant chemotherapy, Treatment interval, Recurrent epithelial ovarian cancer

## Abstract

**Background:**

Secondary cytoreductive surgery (SCS) is possible in selected patients with recurrent epithelial ovarian cancer (EOC). The goal of SCS is complete resection, although chemotherapy is always followed. Delayed intervals between primary debulking surgery and adjuvant chemotherapy was reported to be associated with poorer survivals, however, the role of intervals in recurrent disease is still unknown.

**Materials and methods:**

This retrospective cohort study reviewed data from electronic medical records of women with recurrent EOC treated at Samsung Medical Centre, Seoul, Korea, between January 1, 2002, and December 31, 2015. Patients who underwent SCS with adjuvant chemotherapy for recurrent EOC were eligible. We defined intervals as the period between the day of SCS and the first cycle of adjuvant chemotherapy.

**Results:**

Seventy-nine patients were eligible for this study. Their median age was 48 (range, 18–69) years and median interval between the date of SCS and initiation of adjuvant chemotherapy was 10 (range, 4–115) days. The rate of complete resection was 72.2% (57/79). Division of the patients by interval (Group 1, interval ≤ 10 days; Group 2, interval > 10 days) revealed no difference in clinical parameters. No gross residual disease after SCS (no vs. any gross residual, *p* = 0.002) and longer platinum-free survival (over 12 vs. 6–12 months, *p* = 0.023) were independent favorable prognostic factors in Cox model; however, the intervals did not affect survival.

**Conclusions:**

Delayed intervals to adjuvant chemotherapy after secondary cytoreductive surgery is not associated with decreased survivals. It is important to identify recurrent EOC patients who might have no gross residual disease following SCS. Moreover, surgeons should strive for complete resection.

## Introduction

Epithelial ovarian cancer (EOC) is a deadly disease with a high recurrence rate since most patients are diagnosed with advanced-stage disease [1]. Even with maximal cytoreductive surgery plus platinum-based adjuvant chemotherapy with or without targeted agents, 70–80% of patients who had advanced disease at initial presentation develop recurrent disease within 5 years after these primary standard treatments [2, 3]. Once recurrent disease is confirmed, the goal of treatment is palliative, which is usually managed with systemic therapy [4].

In certain circumstances, secondary cytoreductive surgery (SCS) in selected patients with platinum-sensitive recurrent EOC was reported to provide better survival with acceptable morbidities, although the role of SCS is not yet clearly elucidated [5]. Since palliative systemic therapy is the standard treatment for recurrent EOC, SCS is usually followed by systemic chemotherapy with or without targeted agents.

The intervals between surgery and adjuvant chemotherapy have been investigated due to the concerns that surgery may accelerate residual tumor growth and delayed initiation of adjuvant chemotherapy may lead to poor outcomes [6]. In animal models, removal of the primary tumor enhanced the residual tumor growth; earlier adjuvant chemotherapy reduced this effect, showing better survivals [7]. In patients with solid tumors, this finding has also been observed in various tumor sites including the breast [8] and colon [9]. For ovarian cancers, this topic remains controversial since studies investigating the effect of intervals between maximal cytoreductive surgery and adjuvant chemotherapy are very heterogeneous in terms of patients selection and controlling for bias originated from poor study design [10]. Although a meta-analysis in ovarian cancer suggested a lack of association between intervals and survival in the primary treatment setting [11], some reports have showed negative impacts on survival for delayed intervals in patients with microscopic residuals or residuals of 1–9 mm, suggesting that there may be subsets of patients who may benefit from earlier adjuvant chemotherapy during primary treatment [12–18]. However, it remains unknown whether the intervals between SCS and adjuvant chemotherapy have any effects on survival in recurrent EOC.

## Materials and methods

### Patient selection and data collection

In the present retrospective cohort study, data were reviewed from women with recurrent EOC who were treated at Samsung Medical Centre, Seoul, Korea, between January 1, 2002, and December 31, 2015. Study approval was obtained from the institutional review board (IRB, 2019–03-009). Given the retrospective nature of the study, direct informed consent from the women was not necessary as per the ethical guidelines.

**Fig. 1:**
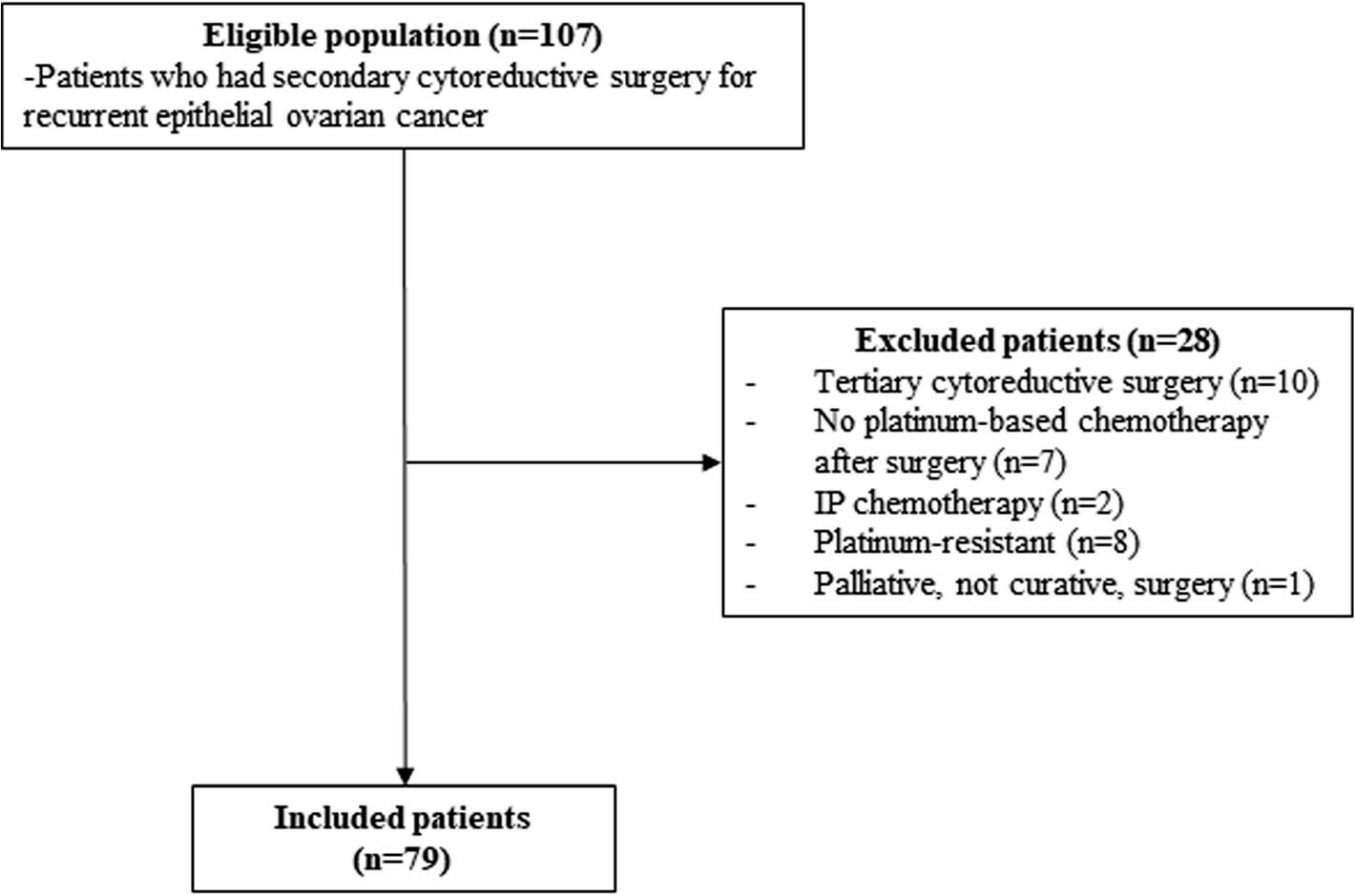
Flow chart of study patients

Eligible patients included those who had SCS and adjuvant chemotherapy in 3 months for recurrent EOC. We considered that there was no intent to adjuvant chemotherapy in patients who started adjuvant chemotherapy beyond this time period. Diagnosis of disease recurrence was made by attending physicians based on images, tumor markers, symptoms and/or biopsies. Although there is no strict consensus among surgeons about the indications for SCS, patients who had isolated or oligometastatic recurrent disease with platinum-free intervals of more than 6 months (ideally more than 12 months) can be considered as candidates for SCS. We excluded patients who underwent tertiary cytoreductive surgery after SCS, did not have adjuvant chemotherapy after SCS, and who received palliative surgery for symptom relief (e.g., end colostomy). The flowchart is shown in Fig. 1. Minimally invasive approaches including laparoscopy or robotic surgery could be used based on the preference of the attending physician. Optimal CRS was defined as a residual disease of less than 10 mm. The data were collected from patient electronic medical records. The interval was defined as the period between the day of SCS and the first day of the first cycle of adjuvant chemotherapy. Platinum-based chemotherapy was usually recommended for these patients.

### Statistical analyses

Statistical analyses were performed with SPSS version 25.0 (SAS Institute, Cary, NC, USA). The descriptive statistics included median (range) for continuous variables (treatment interval, age, cancer antigen 125 [CA 125] level), and number (percentage) for categorical variables (histology, surgery type, American Society of Anaesthesiologists (ASA) score, residual disease, platinum-free interval, number of recurrences). Clinical data were compared by χ2 or Fisher exact tests for categorical variables, and Student’s t- or Wilcoxon rank-sum tests for continuous variables. The primary endpoint was overall survival (OS) and progression-free survival (PFS). Secondary endpoint was perioperative outcomes according to intervals. Also, we validated the Arbeitsgemeinschaft Gynäkologische Onkologie (AGO) score, a model to predict complete resection at SCS, in our study cohort. PFS was defined as the time from SCS to the date of next recurrence/last follow-up. OS was defined as the time from SCS to the date of death/last follow up. OS was calculated by the Kaplan–Meier method. Multivariate analyses were used to evaluate the association of demographic and clinical variables with progression and OS in Cox proportional hazard models. Variables showing a potential association with OS (*p* < 0.05) were selected for multivariate analysis using Cox proportional hazards models and a backward selection algorithm. The criteria for backward selection was *p* < 0.05. Hazard ratios (HRs) were reported with 95% confidence intervals (CIs). Variables such as time-to-treatment interval and CA-125 were analyzed as both continuous and binary variables (dichotomized according to the median value) Two-sided tests were applied. *P*-values < 0.05 were considered statistically significant and were shown up to the thousandths, which were rounded up from the ten-thousandths.

## Results

A total of 79 patients were eligible for this study. Their clinicopathological characteristics are presented in Table 1.

**Table 1:** Patients characteristics by the treatment interval between secondary cytoreductive surgery and adjuvant chemotherapy

	Entire cohort (*n* = 79)	Group 1 (treatment interval ≤ 10 days, *n* = 42)	Group 2 (treatment interval > 10 days, *n* = 37)	*p* value
Treatment interval, days (range)	10 (4–86)	8 (4–10)	19 (11–86)	< 0.001
Median age, yr (range)	48 (18–69)	49 (24–44)	48 (18–69)	0.613
Median CA-125, U/mL (range)	18.4 (1.5–2446.1)	26.1 (1.5–685.2)	15.1 (1.8–2446.1)	0.190
Histology				0.957
Serous	60 (75.9%)	32 (76.2%)	28 (75.7%)	
Non-serous	19 (24.1%)	10 (23.8%)	9 (24.3%)	
Surgery				0.957
Laparoscopy	19 (24.1%)	10 (23.8%)	9 (24.3%)	
Laparotomy	60 (75.9%)	32 (76.2%)	28 (75.7%)	
ASA score^a^				0.798
1	17 (21.5%)	10 (23.8%)	7 (18.9%)	
2	57 (72.2%)	29 (69.0%)	28 (75.7%)	
3	5 (6.3%)	3 (7.1%)	2 (5.4%)	
Residual disease^b^				0.718
R0	57 (72.2%)	29 (69.0%)	28 (75.7%)	
R1	9 (11.4%)	6 (14.3%)	3 (8.1%)	
R2	13 (16.5%)	7 (16.7%)	6 (16.2%)	
Platinum free interval				0.082
6-12mo	14 (17.7%)	10 (23.8%)	4 (10.8%)	
over 12mo	65 (82.3%)	32 (76.2%)	33 (89.2%)	
Recurrence no.				0.243
1st recur	72 (91.1%)	40 (95.2%)	32 (86.5%)	
2nd recur	7 (8.9%)	2 (4.8%)	5 (13.5%)	

^a^ASA score, American Society of Anaesthesiologists score

^b^The level of residual disease after SCS was divided into no gross (R0), 1–9 mm (R1), and equal to or more than 10 mm residual disease (R2)

The median age was 48 (range, 18–69) years and the median CA 125 level before SCS was 18.4 U/mL (range, 1.5–2446.1 U/mL). The dominant histology was high-grade serous carcinoma (60 patients, 75.9%) and 60 patients (75.9%) underwent laparotomy. Most patients (74, 93.7%) had ASA scores of 1–2. The level of residual disease after SCS was divided into no gross residual disease (R0), 1–9 mm residual disease (R1), and equal to or more than 10 mm residual disease (R2). The rate of R0, which was equal to complete resection, was 72.2%. Most patients (65, 82.3%) had a platinum-free interval of more than 12 months and 72 patients (91.1%) had SCS at their first recurrence. All patients received platinum-based chemotherapy and carboplatin with paclitaxel were used in most patients (65, 82.2%). Bevacizumab was added in seven (8.7%) patients.

The overall median interval between the date of SCS and initiation of adjuvant chemotherapy was 10 (range, 4–86) days, and hence, we divided the patients into two groups according to the median interval: Group 1 (interval ≤ 10 days) and Group 2 (interval > 10 days). Groups 1 and 2 were well balanced in terms of clinical parameters, as shown in Table 1. There were no differences between the two groups in procedures and rates of complications during and after SCS (*p* = 0.406, Table 2).

**Table 2:** Surgical procedures and postoperative complications by the treatment interval between secondary cytoreductive surgery and adjuvant chemotherapy

	Entire cohort (*n* = 79)	Group 1 (treatment interval ≤ 10 days, *n* = 42)	Group 2 (treatment interval > 10 days, *n* = 37)	*p* value
Surgical procedures				
Tumorectomy	57 (72.2%)	31 (73.8%)	26 (70.3%)	0.726
LN^a^ dissection	20 (25.3%)	10 (23.8%)	10 (27.0%)	0.743
Bowel surgery	20 (25.3%)	13 (31.0%)	7 (18.9%)	0.220
Bladder/ureter injury	6 (7.6%)	2 (4.8%)	4 (10.8%)	0.311
Vessel injury	2 (2.5%)	2 (4.8%)	0 (0.0%)	0.179
Upper abdomen^b^	13 (16.5%)	9 (21.4%)	4 (10.8%)	0.204
Lung surgery	3 (3.8%)	2 (4.8%)	1 (2.7%)	0.633
Postoperative complications^c^				0.406
I	5 (6.3%)	3 (7.1%)	2 (5.4%)	
II	9 (11.4%)	6 (14.3%)	3 (8.1%)	
III (IIIa, IIIb)	2 (2.5%)	2 (4.8%)	0 (0.0%)	
IV, V	0 (0.0%)	0 (0.0%)	0 (0.0%)	

^a^*LN* Lymph node;

^b^Upper abdomen surgery included splenectomy, pancreatectomy, and hepatectomy

^c^Postoperative complications were classified into ‘Clavien-Dindo classification’

**Fig. 2:**
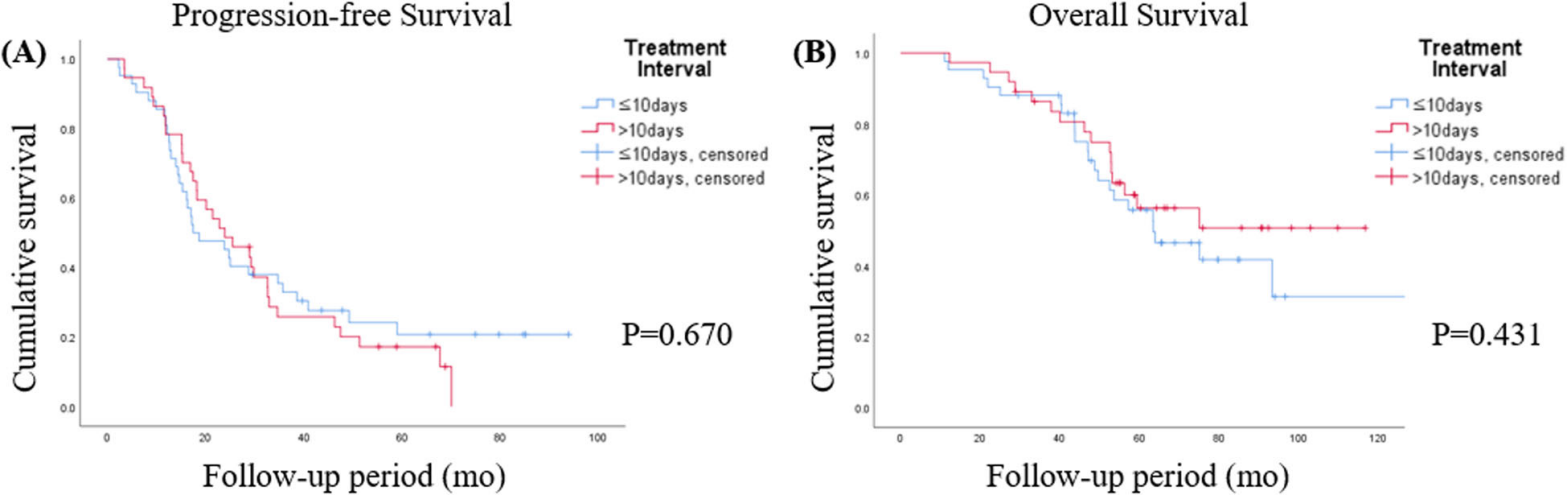
Kaplan–Meier curves of progression-free (**a**) and overall survival (**b**) according to treatment interval

**Fig. 3:**
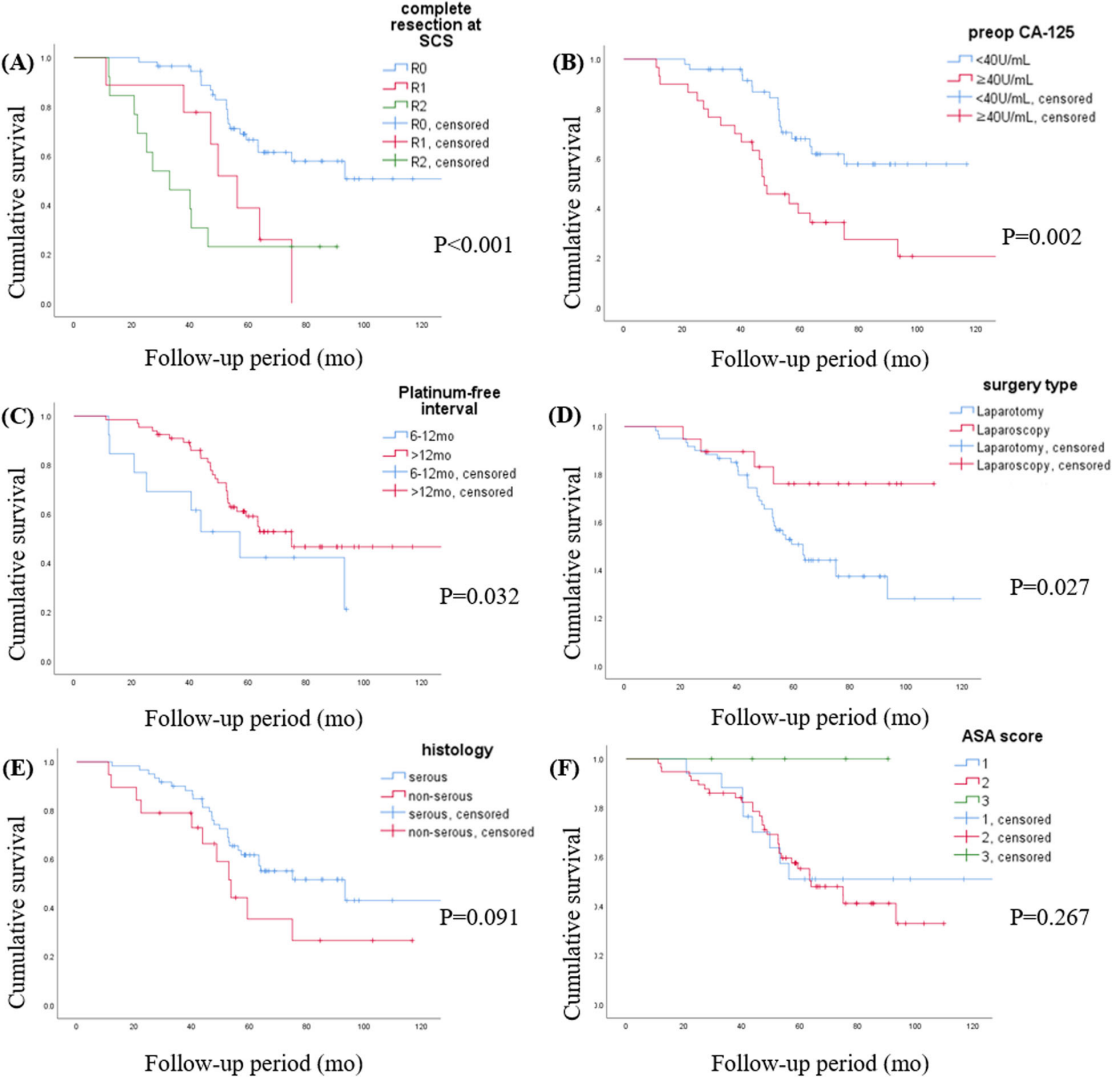
Kaplan–Meier curves of overall survival based on the level of residual disease after SCS (**a**), preoperative cancer antigen 125 (CA-125) levels (**b**), platinum-free interval (**c**), surgery type (**d**), histology (**e**), and American Society of Anaesthesiologists (ASA) score (**f**)

With a median follow-up of 59 months, early chemotherapy (Group 1) after SCS, as opposed to late chemotherapy (Group 2), did not show survival benefits, as shown in Fig. 2a and b. Otherwise, no gross residual disease after SCS, elevation of CA 125 to more than 40 IU/mL before SCS, a platinum-free interval of more than 12 months, laparoscopy during SCS, and serous histology showed significant or marginal better OS, as shown in Fig. 3. A Cox model was used for multivariate analysis of PFS and OS, which showed that the level of residual disease after SCS was the only persistent significant prognostic factor for both. For example, the HR of non-R0 (R1 and R2) over R0 was 2.031 with a 95% CI of 1.161–3.554 (*p* = 0.013, Table 3) for PFS and 3.018 with a 95% CI of 1.486–6.130 (*p* = 0.002, Table 4) for OS. A longer platinum-free interval (over 12 months vs 6–12 months) at the time of SCS was an independent favorable prognostic factor only for OS (HR 0.398; 95% CI: 0.180–0.880; *p* = 0.023). However, the other clinical parameters including the intervals did not affect survival (Tables 3 and 4). When analyzing intervals as a continuous variable, there was still no association between intervals and survivals (HR for PFS, 1.003; 95% CI, 0.984–1.023; *p* = 0.724; HR for OS, 1.002; 95% CI 0.978–1.026; *p* = 0.894).

**Table 3:** Univariate and multivariate Cox Proportional hazard ratios (HR) for disease recurrence

	Univariate		Multivariate	
	HR (95% CI)	*p*-value	HR (95% CI)	*p*-value
Treatment interval^a^				
Group 1	1 (reference)			
Group 2	1.113 (0.680–1.822)	0.670		
Age				
< 65 yr	1 (reference)			
≥ 65 yr	0.679 (0.271–1.701)	0.409		
CA-125				
< 40 U/mL	1 (reference)		1 (reference)	
≥ 40 U/mL	1.976 (1.187–3.287)	0.009	1.620 (0.954–2.749)	0.074
Histology				
Serous	1 (reference)			
Non-serous	1.081 (0.597–1.956)	0.798		
Surgery				
Laparoscopy	1 (reference)			
Laparotomy	1.657 (0.900–3.051)	0.105		
ASA score^b^				
1	1 (reference)			
2, 3	0.808 (0.458–1.426)	0.462		
Residual disease^c^				
R0	1 (reference)		1 (reference)	
R1, R2	2.366 (1.384–4.046)	0.002	2.031 (1.161–3.554)	0.013
Platinum free interval				
6-12mo	1 (reference)		1 (reference)	
over 12mo	0.509 (0.276–0.940)	0.031	0.582 (0.309–1.095)	0.093
Recurrence no.				
1st recur	1 (reference)			
2nd recur	0.967 (0.416–2.247)	0.938		

^a^Enrolled patients were divided into two groups; Group1 (treatment interval ≤ 10 days) and Group2 (treatment interval > 10 days)

^b^ASA score, American Society of Anaesthesiologists score

^c^The level of residual disease after SCS was divided into no gross (R0), 1–9 mm (R1), and equal to or more than 10 mm residual disease (R2)

**Table 4:** Univariate and multivariate Cox Proportional hazard ratios (HR) for death

	Univariate		Multivariate	
	HR (95% CI)	*p*-value	HR (95% CI)	*p*-value
Treatment interval^a^				
Group 1	1 (reference)			
Group 2	0.772 (0.405–1.472)	0.433		
Age				
< 65 yr	1 (reference)			
≥ 65 yr	0.202 (0.028–1.479)	0.115		
CA-125				
< 40 U/mL	1 (reference)		1 (reference)	
≥ 40 U/mL	2.664 (1.401–5.065)	0.003	1.930 (0.997–3.776)	0.054
Histology				
Serous	1 (reference)			
Non-serous	1.820 (0.900–3.682)	0.096		
Surgery				
Laparoscopy	1 (reference)		1 (reference)	
Laparotomy	3.060 (1.081–8.661)	0.035	2.572 (0.889–7.438)	0.081
ASA score^b^				
1	1 (reference)			
2, 3	1.057 (0.484–2.310)	0.889		
Residual disease^c^				
R0	1 (reference)		1 (reference)	
R1, R2	3.699 (1.929–7.093)	< 0.001	3.018 (1.486–6.130)	0.002
Platinum free interval				
6-12mo	1 (reference)		1 (reference)	
over 12mo	0.462 (0.224–0.954)	0.037	0.398 (0.180–0.880)	0.023
Recurrence no.				
1st recur	1 (reference)			
2nd recur	1.277 (0.451–3.619)	0.645		

^a^Enrolled patients were divided into two groups; Group1 (treatment interval ≤ 10 days) and Group2 (treatment interval > 10 days)

^b^ASA score, American Society of Anaesthesiologists score

^c^The level of residual disease after SCS was divided into no gross (R0), 1–9 mm (R1), and equal to or more than 10 mm residual disease (R2)

As AGO score is a validated model to predict complete resection (R0) at SCS in patients with platinum-sensitive recurrent EOC, we applied this model in our study population. Forty-six patients had a positive AGO score and complete resection (R0) was achieved in 37 patients, corresponding to a positive predictive value of 80.4%. In contrast, complete resection (R0) was achieved in 20 of 33 patients with negative AGO scores, resulting in a negative predictive value of 39.4%. Overall, the difference in AGO score (positive vs. negative) before SCS was significantly associated with different rate of residual disease levels after SCS (*p* = 0.047, Table 5).

**Table 5:** Relationship between AGO score^a^ and complete resection at secondary cytoreductive surgery

	Complete resection^b^		Total
	No (R1,R2)	Yes (R0)	
AGO score			
negative	13 (59.1%)	20 (35.1%)	33
positive	9 (40.9%)	37 (64.9%)	46
Total	22	57	79

AGO score assessment had a sensitivity, specificity, positive predictive value, and negative predictive value of 64.9, 59.1, 80.4, and 39.4%, respectively

^a^AGO score, Arbeitsgemeinschaft Gynäkologische Onkologie score

^b^The level of residual disease after SCS was divided into no gross (R0), 1–9 mm (R1), and equal to or more than 10 mm residual disease (R2)

## Discussion

The results of this study found that the intervals between SCS and adjuvant chemotherapy did not influence survival in patients with platinum-sensitive recurrent EOC. Otherwise, patients who had laparotomy, any gross residual disease after SCS, or partially platinum-sensitive recurrence showed poorer survival as compared to those in patients who had a laparoscopy, no gross residual disease after SCS, or platinum-sensitive recurrence.

Preclinical studies have shown that surgery promotes accelerated microscopic or macroscopic residual tumor growth during the perioperative period [6, 18]. The spread of tumor cells during surgery, surgery-induced pro-inflammatory/pro-angiogenic cytokines, and/or transient immune suppression in the immediate postoperative period might be the potential reasons for this phenomenon [6, 19]. Since these negative effects of surgery affect poor oncological outcomes [7, 20, 21], several strategies to overcome these findings have been suggested [6]; among them, animal models showed early chemotherapy after surgery to be an effective way to negate these effects [7, 18]. Concordant with the findings in preclinical studies, clinical studies have demonstrated significantly improved survival with immediate postoperative chemotherapy [9, 19, 22–24]. However, the concept remains controversial and its true impact in cancer patients remains unclear, particularly in EOC. Although immediate postoperative chemotherapy was not associated with poor wound healing in EOC [25], concern remains among gynecologic oncologists.

In a meta-analysis of the impact of intervals between surgery and initiation of adjuvant chemotherapy in the primary treatment of EOC, the intervals were not associated with survival [11]. However, the studies included in the meta-analysis were heterogeneous in terms of the level of residual disease and definitions of early vs. late chemotherapy, which might have influenced the results. For example, Tewari et al. reported that late chemotherapy (> 25 days following surgery) was an independent poor prognostic factor for OS exclusively in patients with no gross residual disease after surgery [15]. The median interval in this study cohort was 31 days (interquartile range, 23–41 days). More recently, we found that late chemotherapy (> 10 days following surgery) was significantly associated with poor OS only in patients with 1–9 mm residual disease [18]. However, late chemotherapy in patients with no gross residual disease did not have any prognostic impact on survival, as opposed to the findings of Tewari et al.; moreover, the median interval of our study was 10 days (range, 3–86 days), significantly shorter than those in the study by Tewari et al. These findings suggest the need for caution when interpreting and applying the results of the meta-analysis to patients.

To obtain more concrete results about the role of intervals on survival in subpopulations of EOC patients, some studies have been performed exclusively in patients with early-stage disease (FIGO I and II) [26], intraperitoneal (IP) chemotherapy [27], or neoadjuvant chemotherapy [16], which found no association between interval and survival. In the same context, studies including only older patients (more than 65 years) [28, 29] or those with high-grade serous ovarian carcinoma [12, 30] showed mixed results regarding the relationship between interval and survival. The association between interval and survival is still unknown in patients with platinum-sensitive recurrent ovarian cancer eligible for SCS. Our findings suggest that delayed chemotherapy following SCS does not influence survival, suggesting that physicians should make their best effort to leave no gross residual disease during SCS, which is one of the most potent prognostic factors in this population, as shown in our and other studies [31].

In our center, SCS was recommended for highly-selected patients based on attending physicians’ decisions; although there was a general consensus (as described in methods), it would be difficult to generalize our results. AGO score is a validated model to predict no gross residual disease after SCS in EOC, with rates of complete resection in the DESKTOP II and III trials with this model of 67% [32] and 75% [33], respectively, comparable to the 72.2% observed in the present study. When we apply AGO score in our study population, the PPV for complete resection of SCS was 80.4%; however, the NPV was relatively low at 39.4%, suggesting there were more patients with negative AGO scores who achieved no gross residual disease during SCS in our study compared to those in previous studies [32, 33]. It is not clear whether this difference originated from recall bias from the retrospective design of our study, which is one of the major limitations, or other factors including different surgical complexity, ethnicity in study population, etc.; nevertheless, we do not think that these differences have influenced our results. However, as the role of intervals is influenced by different definitions of early vs. late chemotherapy and the level of residual disease among various studies based on the primary treatment setting in EOC [10, 11], further studies are needed to draw robust conclusions. Since we did not have a control group of patients who did not receive postoperative chemotherapy after SCS, we cannot determine the role of SCS itself on survival. Recently, SCS in patients with platinum-sensitive first relapsed EOC and a positive AGO-Score resulted in increased PFS in the DESKTOP III trial [33]. OS data in DESKTOP III and the results of surgical parts of the U.S. NRG/Gynecologic Oncology Group (GOG 213) surgical trial are awaited.

One possible explanation for these results is that the study population consisted of patients with small-volume disease before and after SCS. For example, no effect of intervals on survival in FIGO stage I/II (relatively small volume disease as opposed to stage III and IV) at initial presentation or FIGO stage III/IV with no gross residual disease after primary cytoreductive surgery was reported [18, 26]. With longer median intervals (31 days reported by Tewari et al. [15], 19 days reported by Mahner et al. [13]) in more advanced EOC than that observed in our study (10 days), delayed chemotherapy was associated with poorer OS in patients with no gross residual disease after primary cytoreductive surgery, which suggests that our result may have been different if our median intervals were longer. Since the median intervals vary significantly among study populations [10, 11], a multicenter study is warranted.

Apart from the fact that sample size is small, there are still several limitations in this study. First, since it is a retrospective design, unknown factors that might have influenced the intervals may exist even though we found that there was no difference in perioperative outcomes and the rate of complications between early and late adjuvant chemotherapy. Second, it may be hard to generalize our results which came from a single center’s experience for which we need a multicenter study in the future. Third, patients in our cohort may be heterogeneous in terms of tumor burden and sites since indications for SCS was dependent on clinicians’ judgement, not on objective assessment, although AGO score moderately correlated with surgical outcomes in our study population. Despite of these limitations, this is the first study to identify the association between intervals and survivals in recurrent ovarian cancer and can show several messages as follows. Clinicians performing SCS for platinum-sensitive recurrent EOC should select patients in whom complete resection is possible. Adjuvant chemotherapy should be followed at a reasonable interval after SCS in these patients, in whom the treatment goal is usually palliative since there is no evidence that delayed chemotherapy decreased oncological outcomes in this study population.

## Conclusion

In recurrent EOC patients, not only patients’ selection who might have no gross residual disease at SCS is important, but surgeon should also put maximal effort to achieve complete resection. Particularly concerns about possible morbidities during SCS which would delay adjuvant chemotherapy should not justify surgeons to forgo aggressive procedures which may end up incomplete surgery.

## Data Availability

The datasets generated and/or analyzed during the current study are not publicly available due to individual privacy, but are available from the corresponding author on reasonable request.
